# Obstetric and haematological management and outcomes of women with placenta accreta spectrum by planned or urgent delivery: Secondary data analysis of a public referral hospital in Lebanon

**DOI:** 10.1371/journal.pone.0302366

**Published:** 2024-05-08

**Authors:** Stephen J. McCall, Sara Mansour, Janoub Khazaal, Gilles Kayem, Jocelyn DeJong, Rabih Chahine

**Affiliations:** 1 Faculty of Health Sciences, Center for Research on Population and Health, American University of Beirut, Beirut, Lebanon; 2 Department of Obstetrics and Gynecology, Rafik Hariri University Hospital, Beirut, Lebanon; 3 Paris University, Centre for Epidemiology and Statistics Sorbonne Paris Cité (CRESS), Obstetrical Perinatal and Paediatric Epidemiology Research Team, EPOPé, INSERM, Paris, France; 4 Department of Obstetrics and Gynecology, Trousseau Hospital, Assistance Publique-Hôpitaux de Paris, Paris, France; 5 Faculty of Health Sciences, Department of Epidemiology and Population Health, American University of Beirut, Beirut, Lebanon; Ankara Etlik City Hospital, TURKEY

## Abstract

**Background:**

Lebanon has a high caesarean section use and consequently, placenta accreta spectrum (PAS) is becoming more common.

**Objectives:**

To compare maternal characteristics, management, and outcomes of women with PAS by planned or urgent delivery at a major public referral hospital in Lebanon.

**Design:**

Secondary data analysis of prospectively collected data.

**Setting:**

Rafik Hariri University Hospital (public referral hospital), Beirut, Lebanon.

**Participants:**

159 pregnant and postpartum women with confirmed PAS between 2007–2020.

**Main outcome measures:**

Maternal characteristics, management, and maternal and neonatal outcomes.

**Results:**

Out of the 159 women with PAS included, 107 (67.3%) underwent planned caesarean delivery and 52 (32.7%) had urgent delivery. Women who underwent urgent delivery for PAS management were more likely to experience antenatal vaginal bleeding compared to those in the planned group (55.8% vs 28.0%, p<0.001). Median gestational age at delivery was significantly lower for the urgent group compared to the planned (34 vs. 36 weeks, p<0.001). There were no significant differences in terms of blood transfusion rates and major maternal morbidity between the two groups; however, median estimated blood loss was significantly higher for women with urgent delivery (1500ml vs. 1200ml, p = 0.011). Furthermore, the urgent delivery group had a significantly lower birth weight (2177.5g vs. 2560g, p<0.001) with higher rates of neonatal intensive care unit (NICU) admission (53.7% vs 23.8%, p<0.001) and perinatal mortality (18.5% vs 3.8%, p = 0.005).

**Conclusion:**

Urgent delivery among women with PAS is associated with worse maternal and neonatal outcomes compared to the planned approach. Therefore, early referral of women with known or suspected PAS to specialized centres is highly desirable to maximise optimal outcomes for both women and infants.

## Introduction

Placenta accreta spectrum (PAS) is an obstetric condition caused by adherence of the placenta to the uterus [1]. PAS increases the risk of major obstetric haemorrhage, preterm birth, and maternal morbidity and mortality [2]. Over the past three decades, a rising incidence of PAS has been observed globally, which has been primarily linked to the increase in the use of caesarean delivery, a principal risk factor for developing PAS [3–5].

In the Middle East, Lebanon has been witnessing a similar trend of increasing use of caesarean section and this is likely to contribute to a growing incidence of PAS in the country, although to date this relationship has not been explored. The use of caesarean sections has risen from 23% of maternities in 2004 to 40.8% of maternities in 2010 [6]. In 2015, the WHO Eastern Mediterranean Region reported a caesarean birth rate of 46% [7], which is one of the highest in the Arab region [8]. The caesarean section rate further increased from 47% in 2017 to 49.5% in 2019, according to the Ministry of Public Health’s report on caesarean births in Lebanon [9]. Recently, caesarean section was the most common mode of delivery in a tertiary referral hospital in Beirut representing more than 50% of total live births in the period 2018–2020 [10]. PAS is an unintended yet serious consequence of the high use of caesarean section. Furthermore, Lebanese physicians have expressed concern about the emerging rate of abnormal placentation conditions they are observing clinically in the country [11]. In a previous study on maternal ‘near-miss complications’ at a main public referral hospital in Lebanon, 4 out of 5 women who had a maternal ‘near miss’ experienced abnormal placentation, two of which were PAS [11].

In the absence of robust evidence-based standard of care, the optimal management of PAS remains undefined [12]; however, a comprehensive care plan tailored by a multidisciplinary team in a high-resource setting can help guide early diagnosis and timely delivery [13–16]. Scheduled rather than urgent delivery has been shown to reduce haemorrhagic complications and thereby improve maternal as well as neonatal outcomes [15,17–20].

In Lebanon, PAS care is delivered in three specialist referral centres in both private and public settings, with marked disparities in healthcare resources between the two sectors. Within Beirut, Rafik Hariri University Hospital (RHUH) is the only public maternity hospital that provides low-cost and accessible maternal health care for Lebanese and non-Lebanese women. Since the Syrian crisis began in 2011 with the associated influx of refugees to Lebanon, UNHCR-subsidized deliveries in the hospital have increased, and Syrian women now account for the majority of deliveries in that hospital. Due to the high demand for public healthcare especially after the Syrian refugee influx to Lebanon, and in a health system constrained by an economic crisis that began in 2019, delivery of health care for women with PAS in such a resource-limited public setting remains challenging.

Despite the alarmingly high rate of caesarean deliveries at the national level and the severe and life-threatening complications associated with PAS, studies assessing characteristics and outcomes according to planned or urgent approach for PAS management in Lebanon and other Middle Eastern countries are lacking. Therefore, this study aimed to provide evidence on this important maternal health concern, and to compare maternal characteristics, management strategies, and outcomes of women with PAS among those who had planned or urgent delivery at a major public referral hospital in Lebanon.

## Materials and methods

### Study design and setting

This was a secondary data analysis of prospectively collected data at Rafik Hariri University Hospital (RHUH). RHUH is a public and independent hospital, located in Beirut, which offers medical services to Lebanese and non-Lebanese (mainly Syrian refugees) from all regions. In 2012–2013, RHUH was allocated as a key referral centre for PAS diagnosis and management, with a standard care approach and access to a multidisciplinary team including maternal-fetal specialists, surgical expertise (expert gynaecologists, urologists, vascular surgeons), anaesthetists, comprehensive critical care (NICU and adult ICU), and massive transfusion capacity.

The study population was all pregnant and postpartum women who had PAS and delivered in the RHUH between January 2007 and December 2020. Medical records were reviewed by the attending physicians and rotating residents and staff at the Department of Obstetrics and Gynaecology. Anonymized data related to patients’ demographics, clinical characteristics, and outcomes were extracted. For the purposes of this study, the data were accessed in early November 2021 to conduct a secondary analysis.

### Case definition

**Suspected case:** Any pregnant woman with a previous caesarean section and current placenta praevia or low lying at delivery.

**Confirmed case:** A pregnant woman whose placenta is found adherent to the uterine wall and does not separate from the uterine wall during caesarean section or vaginal delivery and, if performed, confirmed by histology of the uterus.

PAS diagnosis was suspected after antenatal imaging (ultrasound, MRI, or both) and then confirmed by the intrapartum findings or histopathological examination after delivery in accordance with the hospital guidelines and practice. Given the paucity or low quality of existing records on women admitted to the hospital, and that many of the women who deliver in the hospital did not receive antenatal care at the out-patient department of the hospital, it is routine practice there to conduct routine ultrasound on all admitted women. Women with antenatal suspicion of PAS but with normal placentation at delivery (i.e., false-positive cases) were excluded from the study. The planned management of PAS was caesarean delivery at 34–35 weeks of gestation. Preoperative preparation included the administration of antenatal steroids and iron. Additionally, single-dose antibiotics prophylaxis was administered unless otherwise indicated. As part of the surgical protocol, uterine incision for caesarean delivery was performed away from the placental bed, followed by the delivery of the fetus and subsequent caesarean hysterectomy.

Haematologic management included preoperative assessment. In cases of massive bleeding, blood transfusions were administered depending on patient’s clinical needs. Commonly administered blood products include packed red blood cells (RBCs), fresh frozen plasma (FFP), and platelets, tailored to individual patient requirements.

### Exposure

Women with PAS included in the study were grouped based on their “urgency of delivery” into either planned or urgent delivery. A delivery was defined as “urgent” in case of maternal or fetal instability including persistent vaginal bleeding, uterine contractions, membrane rupture, preeclampsia, fetal comprise, any signs of labour, or developing maternal comorbidities. Women admitted to the obstetric emergency room or delivery suite at RHUH with any of the aforementioned symptoms, which necessitated an urgent approach, were assigned to the “urgent delivery” group. A delivery was classified as “planned” when it was performed at a time preferred by the mother and the multidisciplinary team in the absence of life-threatening circumstances in both mother and fetus. These included patients at the outpatient department (OPD) who were scheduled for delivery, and were either referred from other centres/private doctors for suspicion of PAS or were followed at RHUH and diagnosed/suspected to have PAS.

### Other variables

All the other variables collected in the database were compared between the two study groups (planned versus urgent) including general and obstetric characteristics, treatment modalities, and clinical outcomes.

*Demographics and obstetrical history* included maternal age, nationality, comorbidities, parity, previous caesarean deliveries, and prior uterine surgery. Maternal comorbidities refer to pre-existing medical conditions present before or during pregnancy and included haematologic disorders, infectious diseases, pregnancy-related complications (gestational diabetes, gestational hypertension, pre-eclampsia), or any history of chronic conditions such as hypertension, diabetes, and asthma.

*Obstetric characteristics* included the first symptom of PAS during pregnancy, placental location, placenta praevia (where the placenta covers the cervical os, or as a low-lying if the placental edge is <2 cm from the cervical os) [21], and antenatal diagnosis (using ultrasound, MRI, or both). Placental invasion (accreta, increta, percreta) into uterine wall was reported based on pathology reports and PAS grading was described following the recently published FIGO Clinical Classification system (only reported since 2019) [22].

*Obstetric and haematologic management* involved surgical methods (total/supracervical hysterectomy or conservative therapy), types of uterine and skin incisions, and blood transfusions needed pre/intra or post-operatively. Conservative therapy refers to all procedures that aim to avoid peripartum hysterectomy, including leaving the placenta in situ. In this study, one case was managed by leaving the placenta in situ and medical treatment with Methotrexate, but this was followed by hysterectomy due to failure of conservative management.

*Maternal outcomes* included maternal death, ICU admission, estimated blood loss, gestational age at delivery, and major morbidities.

*Neonatal outcomes* studied were perinatal mortality, NICU admission, prematurity (defined as any birth before 37 weeks’ gestational age), birth weight, fetal anomalies, and morbidities.

### Statistical methods

Data cleaning and analysis were performed using STATA v15. Descriptive statistics were used to assess maternal characteristics, management, and outcomes in the planned and urgent delivery groups. Continuous variables were tested for normality using histograms and skewness and kurtosis test. Normally distributed continuous variables are summarized as means with standard deviations; skewed continuous variables are reported as medians with interquartile ranges. Categorical variables were presented as frequencies and percentages. For between-group comparisons, categorical variables were analysed using the Chi-square test or Fisher’s exact test when the expected cell count was less than 5. Continuous variables were compared using Student’s t-test or the non-parametric Wilcoxon rank-sum test. Missing data were included as a ‘missing’ category for all categorical variables.

### Ethical approval

This study was approved by Institutional Review Board at the American University of Beirut (BIO-2021-0156) and RHUH ethical review board for the analysis of anonymised data.

## Results

There were 180 pregnant and postpartum women who had at least one previous caesarean section with suspicion PAS during the time-period 2007–2020. A total of 159 women with confirmed PAS were included in this study (n = 157 were confirmed by histology). All women were admitted for delivery at the maternity unit of RHUH between January 2007 and December 2020. The mean age of the sample was 33.7 ± 4.8 years and almost half of the sample consisted of Lebanese women (49.7%); the other half were predominantly Syrian women (44.6%). All women had at least one previous caesarean section and the majority of women had three or more (62.3%). Only 27.9% had a history of other types of uterine surgeries, of which nearly one-fourth were dilatation and curettage procedures (25.9%; Table 1). Most women (69.1%) were diagnosed with placenta praevia in the current pregnancy. More than a third of the women (37.1%) experienced episodes of antenatal vaginal bleeding as their first PAS symptom (Table 2).

**Table 1:** Maternal demographics, obstetrical history, and comorbidities for women with PAS undergoing planned and urgent delivery.

	Total	Planned delivery (n = 107 (67.3%))	Urgent delivery	P-value
	(n = 159)		(n = 52 (32.7%))	
	n (%)	n (%)	n (%)	
**Socio-demographics**				
**Maternal age, y,** *Mean ± SD*	33.7 ± 4.8	33.8 ± 4.9	33.7 ± 4.5	0.946
**Age categories, y**				
<30	28 (17.6)	19 (17.8)	9 (17.3)	0.932
30–34	64 (40.3)	42 (39.2)	22 (42.3)	
≥35	67 (42.1)	46 (43)	21 (40.4)	
**Nationality**				
Lebanese	79 (49.7)	56 (52.3)	23 (44.2)	0.619
Syrian	71 (44.6)	45 (42.1)	26 (50)	
Other	9 (5.7)	6 (5.6)	3 (5.8)	
**Expected primary payer**				
No health coverage	19 (12)	12 (11.3)	7 (13.5)	0.276
Public insurance	72 (45.6)	53 (50)	19 (36.5)	
Private insurance	67 (42.4)	41 (38.7)	26 (50)	
Missing		1	0	
**Maternal medical and obstetrical history**				
**Maternal comorbidities**				
No	132 (83.5)	90 (84.9)	42 (80.8)	0.510
Yes	26 (16.5)	16 (15.1)	10 (19.2)	
Missing		1	0	
**Number of previous caesarean deliveries**				
1	14 (8.8)	8 (7.5)	6 (11.5)	0.409
2	46 (28.9)	34 (31.8)	12 (23.1)	
3 or more	99 (62.3)	65 (60.7)	34 (65.4)	
Duration since last CD*, y, *median (IQR)*	3 (2–5)	2 (2–4)	3.2 (2.2–6)	<0.001
**Parity,** *median (IQR)*	4 (3–5)	4 (3–5)	3.5 (2.5–4.5)	0.394
**Gravidity,** *median (IQR)*	6 (4–7)	5 (4–7)	6 (4.5–7)	0.770
**Other previous uterine surgery**				
0	114 (72.1)	81 (76.4)	33 (63.5)	0.326
1	21 (13.3)	11 (10.4)	10 (19.2)	
2	14 (8.9)	9 (8.5)	5 (9.6)	
3 or more	9 (5.7)	5 (4.7)	4 (7.7)	
Missing		1	0	
**Myomectomy**				
No	155 (98.1)	105 (99.1)	50 (96.2)	0.252
Yes	3 (1.9)	1 (0.9)	2 (3.8)	
Missing		1	0	
**Dilatation & Curettage**				
No	117 (74.1)	82 (77.4)	35 (67.3)	0.176
Yes	41 (25.9)	24 (22.6)	17 (32.7)	
Missing		1	0	
**Other abdominal surgery**				
No	154 (97.5)	104 (98.1)	50 (96.1)	0.599
Yes	4 (2.5)	2 (1.9)	2 (3.9)	
Missing		1	0	

*Missing data for the variable duration since last caesarean delivery (n = 51). Missing data were not included in the percentage.

Abbreviations: PAS, Placenta accreta spectrum; CD, caesarean delivery.

P <0.05 indicates statistical significance.

**Table 2:** Antenatal characteristics, diagnosis, and classification of PAS in women undergoing planned and urgent delivery.

	Total	Planned delivery (n = 107 (67.3%))	Urgent delivery	P-value
	(n = 159)		(n = 52 (32.7%))	
	n (%)	n (%)	n (%)	
**Antepartum characteristics**				
**First Symptom**				
Asymptomatic	91 (57.2)	75 (70.1)	16 (30.8)	<0.001
Vaginal bleeding	59 (37.1)	30 (28)	29 (55.8)	
Labor/abdominal pain	6 (3.8)	2 (1.9)	4 (7.7)	
Membrane rupture	3 (1.9)	0	3 (5.7)	
Gestational age at referral*, week, *median (IQR)*	32 (26–35)	33 (29–36)	28.5 (24–33)	<0.001
**Antepartum haematuria**				
No	157 (98.7)	107 (100)	50 (96.2)	0.106
Yes	2 (1.3)	0	2 (3.8)	
**Placenta praevia**				
No	47 (30.9)	29 (27.9)	18 (37.5)	0.233
Yes	105 (69.1)	75 (72.1)	30 (62.5)	
Missing		3	4	
**Type of placenta praevia** (n = 105)				
Low-lying/marginal/partial	9 (11.2)	5 (8.5)	4 (19)	0.188
Complete	71 (88.8)	54 (91.5)	17 (81)	
Missing		16	9	
**Antenatal diagnosis**				
**Had ultrasound**				
No	1 (0.6)	0	1 (2)	0.320
Yes	152 (99.4)	104 (100)	48 (98)	
Missing		3	3	
**Had MRI**				
No	119 (82.1)	74 (77.1)	45 (91.8)	0.028
Yes	26 (17.9)	22 (22.9)	4 (8.2)	
Missing		11	3	
**Risk assessment on ultrasound**				
Low risk	29 (19.1)	22 (21.2)	7 (14.6)	0.233
Moderate risk	8 (5.3)	3 (2.9)	5 (10.4)	
High risk	76 (50)	51 (49)	25 (52.1)	
Risk not assessed	39 (25.6)	28 (26.9)	11 (22.9)	
**Risk assessment on MRI**				
Evidence of abnormal placentation	14 (53.8)	13 (59.1)	1 (25)	0.383
Low suspicion	1 (3.9)	1 (4.6)	0	
High suspicion	9 (34.6)	6 (27.3)	3 (75)	
Missing		2	0	
**Grading and depth of placental invasion**				
**FIGO Clinical classification**				
Grade 1	4 (8.7)	3 (8.3)	1 (10)	0.999
Grade 2	18 (39.1)	14 (38.9)	4 (40)	
Grade 3a	23 (50)	18 (50)	5 (50)	
Grade 3b	1 (2.2)	1 (2.8)	0	
Missing		71	42	
**Placental pathology**				
Accreta	38 (27.9)	22 (23.7)	16 (37.2)	0.031
Increta	79 (58.1)	61 (65.6)	18 (41.9)	
Percreta	19 (14)	10 (10.7)	9 (20.9)	
Missing		14	9	

*Missing data for the variable gestational age at referral (n = 6). Missing data were not included in the percentages.

Abbreviations: PAS, Placenta accreta spectrum; MRI, Magnetic Resonance Imaging.

P <0.05 indicates statistical significance.

**Fig 1:**
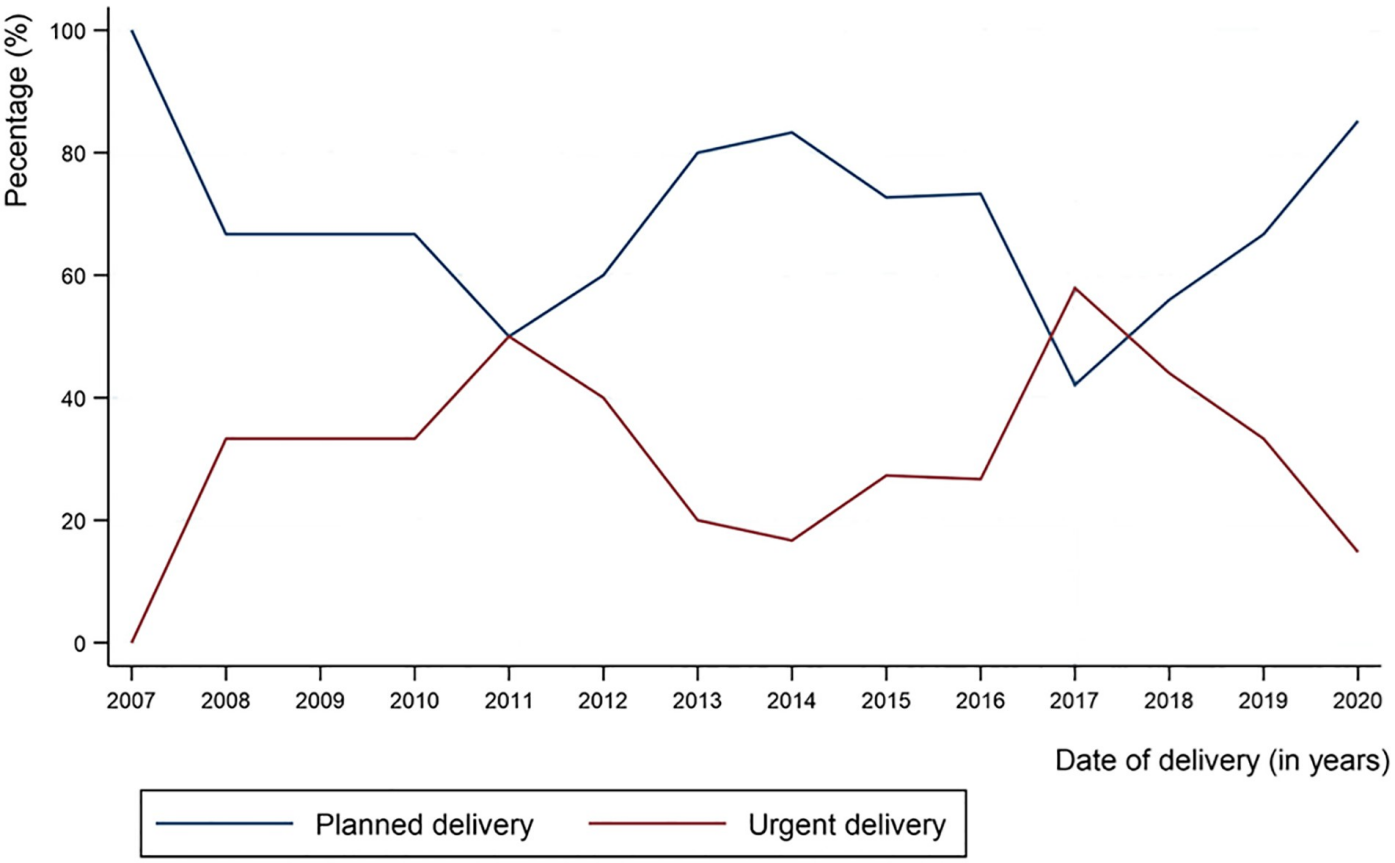
Rate of planned versus urgent delivery for women with PAS admitted to RHUH by year, 2007–2020.

Out of the total, 67.3% of women underwent a planned caesarean delivery and 32.7% had an urgent caesarean delivery. The rates of planned and urgent deliveries have varied over the period 2007 to 2020 as shown in Fig 1. For the comparisons between planned and urgent delivery groups, there were no significant differences in terms of the general maternal demographic characteristics and obstetrical history except that the women in the urgent delivery group had significantly longer intervals since their last caesarean section (3.2 years vs. 2 years, p<0.001; Table 1).

Compared with the planned delivery group, a significantly higher proportion of women in the urgent delivery group experienced antepartum haemorrhage (55.8% vs 28%, p<0.001). Women in the planned delivery group had a higher proportion of placenta increta (65.6% vs 41.9%) and a lower proportion of placenta accreta (23.7% vs 37.2%, p = 0.031; Table 2).

For obstetric management, there were no significant differences between the study groups concerning the treatment and surgical modalities (Table 3). As for haematologic management, median preoperative haemoglobin was significantly lower in the urgent group (10.8 g/dl, range 9.2–11.9) compared to the planned group (11.3 g/dl, range 10.3–12.3, p<0.001). No other significant differences were seen in the timing of blood transfusions and the number of blood units transfused between women who had planned versus urgent delivery (Table 3).

**Table 3:** Obstetric and haematologic management of PAS in women undergoing planned and urgent delivery.

	Total	Planned delivery (n = 107 (67.3%))	Urgent delivery	P-value
	(n = 159)		(n = 52 (32.7%))	
	n (%)	n (%)	n (%)	
**Obstetric management**				
**Conservative management**				
No	157 (99.4)	106 (100)	51 (98.1)	0.329
Yes	1 (0.6)	0	1 (1.9)	
Missing		1	0	
**Had hysterectomy**				
No	1 (0.6)	1 (0.9)	0	0.999
Yes	158 (99.4)	106 (99.1)	52 (100)	
**Hysterectomy, type**				
Subtotal/supracervical	35 (22.1)	25 (23.6)	10 (19.2)	0.536
Total	123 (77.9)	81 (76.4)	42 (80.8)	
**Vertical skin incision**				
No	23 (14.5)	15 (14)	8 (15.4)	0.818
Yes	136 (85.5)	92 (86)	44 (84.6)	
**Type of anaesthesia**				
Spinal/regional	10 (6.3)	8 (7.5)	2 (3.8)	0.073
General	25 (15.7)	12 (11.2)	13 (25)	
Both	124 (78)	87 (81.3)	37 (71.2)	
**Operative time, in minutes,** *median (IQR)*	140 (120–180)	140 (120–180)	137.5 (120–172.2)	0.483
**Haematologic management**				
**Preoperative haemoglobin, g/dl,** *median (IQR)*	11.2 (10.3–12.1)	11.3 (10.3–12.3)	10.8 (9.2–11.9)	<0.001
**Postoperative haemoglobin, g/dl,** *median (IQR)*	10.4 (9.1–11.3)	10.7 (9.2–11.5)	10 (9–11.1)	0.110
**Blood transfusion**				
No	43 (27)	33 (30.8)	10 (19.2)	0.122
Yes	116 (73)	74 (69.2)	42 (80.8)	
**Timing of transfusion**				
Preoperative transfusion	1 (0.9)	0	1 (2.4)	0.455
Post/intra-operative transfusion	99 (85.3)	63 (85.1)	36 (85.7)	
Both	16 (13.8)	11 (14.9)	5 (11.9)	
**Blood products received preoperatively**				
Packed Red blood cells (PRBCs)				
Yes	17 (100)	11 (100)	6 (100)	-
Fresh frozen plasma (FFP)				
No	13 (76.5)	8 (72.7)	5 (83.3)	0.999
Yes	4 (23.5)	3 (27.3)	1 (16.7)	
Red blood cells units transfused, *median (IQR****)***	3 (2–3)	2 (2–3)	3 (2–5)	0.276
Fresh frozen plasma units transfused, *median (IQR****)***	3.5 (2.5–5)	4 (3–6)	2 (2–2)	0.179
**Blood products received intra/post operatively**				
Packed Red blood cells (PRBCs)				
Yes	115 (100)	74 (100)	41 (100)	-
Fresh frozen plasma (FFP)				
No	57 (49.6)	41 (55.4)	16 (39)	0.092
Yes	58 (50.4)	33 (44.6)	25 (61)	
Platelets				
No	110 (95.7)	71 (96)	39 (95.1)	0.999
Yes	5 (4.3)	3 (4)	2 (4.9)	
Whole blood				
No	114 (99.1)	73 (98.7)	41 (100)	0.999
Yes	1 (0.9)	1 (1.3)	0	
Red blood cells units transfused, *median (IQR****)***	3 (2–6)	3 (2–6)	4 (2–6)	0.113
Fresh frozen plasma units transfused, *median (IQR****)***	4 (2–4)	4 (2–4)	4 (2–4)	0.987
Platelet units transfused, *median (IQR****)***	2 (1–2)	2 (1–3)	1.5 (1–2)	0.591
**Total units of transfused blood products (pre and intra/postoperatively)**				
Total units of RBCs transfused, *median (IQR****)***	4 (2–6)	3 (2–6)	4 (2–6)	0.352
Total units of FFP transfused, *median (IQR****)***	4 (2–5)	4 (2–6)	4 (2–5)	0.641

Missing data were not included in the percentages.

Abbreviations: RBC, red blood cells; FFP, fresh frozen plasma.

P <0.05 indicates statistical significance.

Urgent delivery occurred at significantly lower median gestational ages (34 weeks vs 36 weeks, p<0.001). Regarding maternal outcomes, the median estimated blood loss was significantly higher in women who required an urgent delivery (1500ml, interquartile range 1200-2000ml) as compared to a planned delivery (1200ml, range 800-2000ml, p = 0.011; Table 4). Women in the urgent delivery group did not experience higher rates of major maternal morbidities or post-operative complications, including organ injury. Similarly, there were no significant differences in the rates of ICU admissions, duration of hospital stay, or maternal death between the two groups (Table 4).

**Table 4:** Maternal outcomes of women with PAS undergoing planned and urgent delivery.

	Total	Planned delivery (n = 107 (67.3%))	Urgent delivery	P-value
	(n = 159)		(n = 52 (32.7%))	
	n (%)	n (%)	n (%)	
**Maternal outcomes**				
**Maternal death**				
No	150 (99.3)	101 (100)	49 (98)	0.331
Yes	1 (0.7)	0	1 (2)	
Missing		6	2	
**ICU admission**				
No	149 (93.7)	102 (95.3)	47 (90.4)	0.297
Yes	10 (6.3)	5 (4.7)	5 (9.6)	
Duration of ICU stay*, days, *median (IQR****)***	2.5 (1–4.5)	2 (1–3)	3 (1–6)	0.758
**Postpartum haemorrhage (≥1000 ml)**				
No	93 (58.5)	65 (60.8)	28 (53.9)	0.407
Yes	66 (41.5)	42 (39.2)	24 (46.1)	
Estimated blood loss*, ml, *median (IQR****)***	1500 (1000–2000)	1200 (800–2000)	1500 (1200–2000)	0.011
**Gestational age at delivery,** week, *median (IQR****)***	35 (34–36)	36 (35–36)	34 (28.5–35)	<0.001
Total hospital stay*, days, *median (IQR****)***	5 (4–7)	5 (3–7)	5 (4–7)	0.476
**Major maternal morbidity**				
Organ injury (bladder, bowel, ureters)				
No	108 (67.9)	74 (69.2)	34 (65.4)	0.632
Yes	51 (32.1)	33 (30.8)	18 (34.6)	
Thrombotic event				
No	157 (98.7)	106 (99.1)	51 (98.1)	0.549
Yes	2 (1.3)	1 (0.9)	1 (1.9)	
Wound infection				
No	157 (98.7)	107 (100)	50 (96.1)	0.106
Yes	2 (1.3)	0	2 (3.9)	
Cardiac arrest				
No	158 (99.4)	106 (99.1)	52 (100)	0.999
Yes	1 (0.6)	1 (0.9)	0	
Pelvic haematoma				
No	156 (98.1)	105 (98.1)	51 (98.1)	0.999
Yes	3 (1.9)	2 (1.9)	1 (1.9)	
Genitourinary fistula				
No	157 (98.7)	106 (99.1)	51 (98.1)	0.549
Yes	2 (1.3)	1 (0.9)	1 (1.9)	
Uterine rupture				
No	158 (99.4)	107 (100)	51 (98.1)	0.327
Yes	1 (0.6)	0	1 (1.9)	

*Missing data for the following variables: Duration of ICU stay (n = 2), EBL (n = 3), ICU admission (n = 2), Total hospital stay (n = 9).

Missing data were not included in the percentages.

Abbreviations: PAS, placenta accreta spectrum; ICU, intensive care unit.

Neonates of women who underwent urgent delivery had lower birth weights (2177.5g vs 2560g, p<0.001), were more likely to be admitted to neonatal intensive care units (53.7% vs 23.8%, p<0.001), and had significantly higher proportions of perinatal mortality (18.5% vs 3.8%, p = 0.005) compared to those of women who had planned delivery (Table 5).

**Table 5:** Neonatal outcomes of women with PAS undergoing planned and urgent delivery.

	Total	Planned delivery (n = 108 (66.7%))	Urgent delivery	P-value
	(n = 162)		(n = 54 (33.3%))	
	n (%)	n (%)	n (%)	
**Neonatal outcomes**				
**Gestational age at birth (weeks)**				
<34 weeks	33 (20.4)	7 (6.5)	26 (48.2)	
34–37 weeks	114 (70.4)	90 (83.3)	24 (44.4)	<0.001
>37 weeks	15 (9.2)	11 (10.2)	4 (7.4)	
Birthweight*, g, *median (IQR****)***	2440 (2145–2825)	2560 (2215–2885)	2177.5 (1490–2535)	<0.001
**Perinatal mortality**				
No	146 (91.2)	102(96.2)	44(81.5)	0.005
Yes	14 (8.8)	4(3.8)	10(18.5)	
Missing		2	0	
**Type of perinatal mortality (n = 14)**				
Stillbirth/ Intrauterine fetal death (IUFD)	7 (50)	3 (75)	4 (40)	0.237
Neonatal death	7 (50)	1 (25)	6 (60)	
**NICU admission**				
No	105 (66)	80 (76.2)	25 (46.3)	<0.001
Yes	54 (34)	25 (23.8)	29 (53.7)	
Missing		3	0	
**Fetal anomalies**				
No	146 (91.2)	97 (91.5)	49 (90.7)	0.999
Yes	14 (8.8)	9 (8.5)	5 (9.3)	
Missing		2	0	
**Neonatal morbidities**				
Respiratory distress syndrome	12 (8.6)	10 (10.1)	2 (4.9)	0.509
Severe infection (e.g. sepsis)	2 (1.4)	1 (1)	1 (2.4)	0.501
Transient tachypnoea	1 (0.7)	1 (1)	0	0.999
Hyperbilirubinemia	2 (1.4)	2 (2)	0	0.999
Hypoglycaemia	1 (0.7)	1 (1)	0	0.999
Missing		9	13	

*Missing data for the variable birthweight (n = 6). Missing data were not included in the percentages.

Abbreviations: PAS, placenta accreta spectrum; NICU, neonatal intensive care unit.

## Discussion

### Main findings

Among the 159 women with PAS, 67% of the study population had a planned delivery. Women who had an urgent delivery for PAS had similar characteristics and outcomes compared to women with a planned delivery other than a higher median blood loss. However, infants born to women with an urgent delivery had poorer outcomes than infants born from women with a planned delivery. These infants were more likely to be of lower birth weight, admitted to NICU, and had a higher likelihood of perinatal mortality.

### Results in context

#### Antenatal characteristics and diagnosis

Similar to our findings, previous studies have found that antenatal haemorrhage was more common for women with urgent delivery than planned delivery [18,23–25]. This could explain our finding of the lower levels of preoperative haemoglobin in women who delivered urgently, which may relate to the antenatal bleeding experienced by these women. Referral to tertiary centres after antenatal bleeding allowed PAS or other maternal complications to be detected and enabled preparation for high-risk pregnancy in well-resourced facilities before the onset of labour.

Women with the least placental invasion (accreta) were more likely to undergo an urgent rather than planned delivery, which is consistent with findings of previous studies [18,26,27]. A possible explanation of this may be attributed to the fact that milder degrees of placental invasion can go undetected on ultrasounds. Ultrasound detection of PAS is based on clinician experience and is a suspicion until confirmed [27]. This might have prevented the early detection of minor placental invasions in our study, especially as a proportion of patients were referred from different centres with limited experience in PAS. Additionally, patients referred to RHUH often arrive late, with little or no antenatal care and with major haemorrhage. This is likely to have contributed to urgent deliveries and limited the opportunity for planned interventions.

#### Management and outcomes

Urgent delivery of women with PAS was associated with adverse maternal outcomes in terms of increased maternal blood loss in this study. Comparable findings have been reported in an international multicentre study by Schwickert et al. [28] which demonstrated an increased likelihood of median blood loss >3500mL in unplanned compared to planned delivery among 338 women with PAS. However, it should be noted that the median blood loss for the urgent PAS delivery group in our study was low compared to other studies ranging from 2400 ml to >5000 ml [25,28–31]. This can be supported by the finding of a previous study where the presence of a PAS treatment team with considerable surgical expertise had the strongest correlation with reduced odds of maternal blood loss [28,32]. RHUH has an experienced multidisciplinary team that regularly performs complex surgeries for PAS, which may account for the lower maternal blood loss observed with urgent delivery.

Our results are in agreement with Morlando et al. [18], which found no significant differences in transfusion rates or major morbidity between planned and emergency deliveries among women with PAS. On the contrary, a systematic review and meta-analysis of nine related studies comparing maternal outcomes for PAS patients delivering via planned and emergency procedures showed that women undergoing planned delivery required fewer units of transfused blood, experienced shorter hospital stay durations, and presented reduced risks for maternal ICU admission and severe maternal morbidity [20]. The review results may be explained by differences in levels of experience of clinicians in PAS management. Considering the heterogeneity of PAS and the complexity of managing accreta cases, maternal outcomes greatly depend upon the skills and competency of clinical teams involved [32]. In the same centre, maternal outcomes were found to improve with the cumulative experience of clinical teams [33]. Also, hysterectomies performed by inexperienced surgeons were shown to be associated with increased rates of complication, transfusion, and mortality compared with experienced surgeons [34]. In RHUH, the established PAS team has gained advanced expertise in the medical and surgical management of PAS over the years, which may explain the lack of difference across both delivery groups.

Infants born to women with an urgent delivery for PAS had worse clinical outcomes including lower gestational age, higher NICU admission, and perinatal mortality. This is consistent with the findings of previous research where a planned delivery for PAS with a multidisciplinary team showed favourable neonatal outcomes when compared with an urgent delivery [18,20,23,35]. However, it is worth noting that several studies did not find significant differences in neonatal outcomes between the two delivery groups [25,36,37]. The most relevant factor influencing neonatal outcomes in women with PAS is gestational age at delivery, with higher gestational ages leading to improved neonatal outcomes [18,38]. Given that urgent delivery often occurs at lower gestational ages in pregnancies complicated by PAS, this can explain the reduced birth weight that is often accompanied by NICU admissions and mortality.

All women with a previous caesarean section and a low-lying placenta should be considered high risk for PAS; these should be referred and managed at a tertiary hospital. The introduction of a coordinated multidisciplinary approach at this public referral hospital has contributed to a reduced rate of urgent deliveries and allowed for an increased scheduling of deliveries in women with PAS. Several studies have emphasized the role of multidisciplinary care in improving outcomes and reducing both maternal and neonatal morbidity and mortality [2,13,14,31,32,39]. In Lebanon and other lower-middle income countries, the management of PAS in resource-limited settings is still challenging [26,40]. As such, PAS management strategies need to be adapted to local needs to optimize outcomes for both women and infants.

At a larger health system level, the paper provides evidence that substantiates concerns raised by physicians in Lebanon about the medical consequences for women and their newborns of high use of caesarean section delivery in the country. Managing placental complications represents a significant use of specialized clinicians in this public maternity facility in a lower-middle income country experiencing economic crisis and that has one of the highest refugee to host population ratios in the world. Moreover, the consequences of the associated NICU admission pose particularly heavy costs, in a context of a health sector losing human resources–including highly trained NICU staff–due to the deterioration in the purchasing power of health provider salaries.

#### Strengths and limitations

Our study is the first study to address the characteristics and outcomes of women with PAS population in Lebanon. Nearly all PAS cases were histologically confirmed (n = 157/ n = 159), which reduced the likelihood of inclusion of false-positive cases.

This study was limited by its relatively small sample size which may not have been sufficient to detect statistically significant differences between the planned and urgent delivery groups. This study had a small amount of missing data, which may be related to poor documentation from women who were referred from private clinics or hospitals. Lastly, this study was a single centre experience and its findings as it may not be representative of the general PAS population in Lebanon.

## Conclusion

PAS requiring urgent delivery was associated with adverse neonatal outcomes. Considering that Lebanon has a relatively high rate of caesarean section, multi-faceted efforts should be directed to diagnosing women at high-risk for PAS at the earliest opportunity. This allows for better PAS management at experienced referral centres with an aim to reduce related morbidity and mortality. Future larger population-based studies are required in Lebanon to provide us with a comprehensive understanding of PAS in the country.

## Supporting information

S1 File STROBE checklist. (DOCX)

S2 File PAS surgical technique. (DOCX)
