# Supplementary material for: Obstetric and haematological management and outcomes of women with placenta accreta spectrum by planned or urgent delivery: Secondary data analysis of a public referral hospital in Lebanon
Source: PLoS One. 2024 May 8;19(5):e0302366. doi: 10.1371/journal.pone.0302366 (PMC11078361; doi:10.1371/journal.pone.0302366)
Supplement: S2 File — (DOCX) [file pone.0302366.s002.docx]

**Surgical approach to PAS**

Delivery planned 34-35 weeks

Preparation with antenatal steroids for fetal lung maturation, and maternal iron administration to optimize pre- operative hemoglobin for better surgical outcome and patient blood management

Single dose antibiotics prophylaxis (unless indicated otherwise)

1. Midline abdominal incision and release of all possible pelvic-abdominal adhesion to clear the surgical field.
2. Intra- op inspection: in case of confirmed PAS, proceed with fundal uterine incision and delivery of the baby
3. “NO TRACTION ON THE PLACENTA”, the umbilical Cord is ligated, and the uterine incision is closed primarily with placenta in situ
4. Proceed for hysterectomy
5. Round ligament and infandibulo-pelvic ligament and vessels - preservation of the ovaries.
6. Open anterior peritoneum and start dissecting the bladder **lateral to medial on the uterus while avoiding the most adherent parts of it with the placenta.** Care is taken to avoid surgically perforating or rupturing the very thin vesico- placenta surface.
7. Open the peritoneum posteriorly, identify the ureters and the extent of placental invasion to the uterine parametrial region, dissect the ureters away and down. This is done concomitantly or in alternation with step 6.
8. End up isolating the most adherent part of the bladder as medial as possible, this will allow for clamping and suture ligation of the uterine vessels as safely as possible in case of incident bleeding or placental rupture prematurely ( prior to final few steps before uterine removal)
9. Complete bladder dissection caudally until totally free from placenta.
10. Identify the lower border of the placenta, this will demarcate the limit of clamping upon hysterectomy, in case a subtotal hysterectomy is decided.
11. Proceed with suture ligation and cutting of the uterine arteries and cardinal ligaments down until the lower border of the placenta is reached, then the uterus is removed “en bloc with Placenta” in the usual manner above transverse clamps pre-positioned at the vaginal vault area (sometimes cervical in case of subtotal hysterectomy).
12. Hemostasis is secured : 2 paramount areas

- Lateral clamps of the uterine vessels
- Vesico-uterine service and possible missed bridging vessels

1. The vault is sutured and closed taking care of avoiding the bladder borders
2. The bladder integrity is checked primarily then after filling with saline to ensure no break of bladder walls. Ureters re- explored and their integrity as well as their path to the bladder tunnel away from any sutures or ligation is ensured.
3. Final inspection for hemostasis, integrity of all intra-abdominal organs and final surgical count done and declared
4. Closure of the abdomen in the usual manner
5. Patient taken to recovery room – HDU (high dependency unit) for post- operative monitoring
